# Sex-Specific Differences in Running Injuries: A Systematic Review with Meta-Analysis and Meta-Regression

**DOI:** 10.1007/s40279-020-01412-7

**Published:** 2021-01-12

**Authors:** Karsten Hollander, Anna Lina Rahlf, Jan Wilke, Christopher Edler, Simon Steib, Astrid Junge, Astrid Zech

**Affiliations:** 1 Medical School Hamburg, Hamburg, Germany; 2 Department of Physical Medicine and Rehabilitation, Spaulding National Running Center, Harvard Medical School, Cambridge, MA USA; 3 Department of Human Movement Science and Exercise Physiology, Institute of Sport Science, Friedrich Schiller University Jena, Jena, Germany; 4 Department of Sports Medicine and Exercise Physiology, Goethe University Frankfurt, Frankfurt, Germany; 5 Prevention, Rehabilitation and Interdisciplinary Sports Medicine, BG Trauma Hospital of Hamburg, Hamburg, Germany; 6 Department of Human Movement, Training and Active Aging, Institute of Sports and Sports Science, Heidelberg University, Heidelberg, Germany; 7 Swiss Concussion Center, Schulthess Klinik, Zürich, Switzerland

## Abstract

**Background:**

Running is a popular sport with high injury rates. Although risk factors have intensively been investigated, synthesized knowledge about the differences in injury rates of female and male runners is scarce.

**Objective:**

To systematically investigate the differences in injury rates and characteristics between female and male runners.

**Methods:**

Database searches (PubMed, Web of Science, PEDro, SPORTDiscus) were conducted according to PRISMA guidelines using the keywords “running AND injur*”. Prospective studies reporting running related injury rates for both sexes were included. A random-effects meta-analysis was used to pool the risk ratios (RR) for the occurrence of injuries in female vs. male runners. Potential moderators (effect modifiers) were analysed using meta-regression.

**Results:**

After removal of duplicates, 12,215 articles were screened. Thirty-eight studies were included and the OR of 31 could be pooled in the quantitative analysis. The overall injury rate was 20.8 (95% CI 19.9–21.7) injuries per 100 female runners and 20.4 (95% CI 19.7–21.1) injuries per 100 male runners. Meta-analysis revealed no differences between sexes for overall injuries reported per 100 runners (RR 0.99, 95% CI 0.90–1.10, *n* = 24) and per hours or athlete exposure (RR 0.94, 95% CI 0.69–1.27, *n* = 6). Female sex was associated with a more frequent occurrence of bone stress injury (RR (for males) 0.52, 95% CI 0.36–0.76, *n* = 5) while male runners had higher risk for Achilles tendinopathies (RR 1. 86, 95% CI 1.25–2.79, *n* = 2). Meta-regression showed an association between a higher injury risk and competition distances of 10 km and shorter in female runners (RR 1.08, 95% CI 1.00–1.69).

**Conclusion:**

Differences between female and male runners in specific injury diagnoses should be considered in the development of individualised and sex-specific prevention and rehabilitation strategies to manage running-related injuries.

**Supplementary Information:**

The online version contains supplementary material available at 10.1007/s40279-020-01412-7.

## Key Points

**Table:** 

There were no differences between female and male runners in overall injury rates.
Female runners had more bone stress injuries.
Male runners had more Achilles tendon injuries.
Shorter competition distances increase the risk of injury for female runners.

## Introduction

Running is a very popular sport practiced all over the world. While regular physical activity and sports such as running are beneficial for prevention and rehabilitation of many health complaints (“exercise is medicine”) [1, 2], running is frequently associated with high injury prevalence and incidence rates [3–5].

For injury prevention, risk factors need to be well understood [6]. Risk factors for running are manifold and consist of training load, biomechanical, anatomical and anthropometrical variables [7–12]. While some previous studies exclusively investigated either male [9, 13] or female [14–16] runners, sex has been suggested to be a risk factor for specific injury patterns in running, as well as for overall injury risk [7, 17, 18]. This is supported by a study investigating injury rates for female and male elite running athletes [19]. Analysing data collected during 14 international athletics championships, Edouard et al. [19] showed that male elite athletes had lower injury incidence rates for bone stress injuries (BSI) than female counterparts. However, injury risks differed between sexes for running disciplines (from middle distances upwards) although only with a small to trivial relative risk (1.5 for middle distances, 0.9 for long distances and 1.3 for marathon running) [19].

Including and investigating both sexes in running injury research is in line with evidence for the different risks between female and male athletes for specific types of injuries such as anterior cruciate ligament ruptures or concussions in different team sports as well as ankle sprains in all sports [20–22]. However, considering the current literature, it is difficult to derive conclusive summaries about differences in overall or specific injury epidemiology for both sexes in specific sports [18]. To develop and optimize individualized prevention and treatment options for running injuries, it is crucial to understand if and to what extent injury epidemiology differs between the sexes. Therefore, the aim of this systematic review was to evaluate the differences in injury rates and characteristics between male and female runners using meta-analytical techniques. First, differences in overall injury rates were compared between both sexes. Secondly, depending on the availability of sufficient data, specific injury diagnoses were analysed regarding their occurrence in female and male runners.

## Methods

This study was conducted and presented according to the PRISMA guidelines for reporting systematic reviews and meta-analysis [23]. Prior to the start of the study, the review protocol was registered in the PROSPERO database (CRD4201911883).

### Search Strategy and Inclusion Criteria

Two independent investigators (K.H. and C.E.) conducted a systematic literature search including articles from inception till April 2020. Prospective cohort studies and randomized controlled trials investigating healthy runners from different age groups were included. The search was restricted to articles from peer-reviewed journals published in English, German, or Spanish languages. Furthermore, studies had to report rates of running-related injuries for both sexes. Overall injury rates and injury rates for specific locations, diagnoses or injury mechanisms were considered. Included running disciplines were middle distance and long-distance track as well as cross-country, trail and road running. There was no restriction to a specific injury definition. Reviews, systematic reviews, commentaries, case studies, case series, cross-sectional studies, retrospective studies and interventional arms of randomised controlled trials (RCT) were excluded. For RCTs, only untreated control groups were considered.

The search strategy using specific keywords (running AND injur*) was applied to four different databases (PubMed, Web of Science, PEDro, SPORTDiscus). All databases were searched to identify relevant studies based on keywords, title and abstract. Two independent investigators (C.E. and K.H.) extracted relevant studies based on the inclusion criteria first by reading the title, the abstract and the full text, if available. A third reviewer (A.Z.) was available for consensus decisions. The bibliographical information of included articles was examined for further relevant references (backward search). A forward search was done via citation tracking using Web of Science® (Thomson Reuters).

### Data Extraction

Study characteristics (design, running discipline, population, age and number of participants) as well as prevalence and incidence rates for both sexes were extracted. For prevalence rates, number of injuries or number of injured runners were related to the number of runners investigated. For incidence rates, number of injuries and specific exposures (in hours, kilometres or athlete exposure) were used. An athlete exposure (AE) is defined as one athlete participating in one practice or competition [24]. When it was not possible to extract the data from an article for specific running distances (e.g., pooling of overall injuries for track disciplines), corresponding authors were contacted by email to obtain the data. If specific data were not able to be obtained, the respective study was included in the systematic review but not in subsequent analyses.

### Study Quality Assessment

Due to insufficient study quality assessment tools in sports injury epidemiology, a new tool was developed by consensus of K.H., A.J., A.L.R., A.Z. and S.S. on the basis of previously used tools [20, 22, 25, 26]. The modification ensured that all relevant points regarding the quality of the study design and important content-related information would be considered—e.g., differences in methodological approaches such as competition or season, or the type of data collection.

This tool consisted of 15 items on recruitment, reporting, injury and exposure collection, injury definition and drop-out (Table 1).

**Table 1:** Risk of bias assessment tool

Question	Rating
Are the sources and methods of participant recruitment clearly described?	Yes (1), no (0)
Are the relevant characteristics (n, age, sex, sport, level of competition) of the study population reported?	Yes (1), no (0)
Does the study cover season and/or tournaments/championships?	Season (2), tournaments (1), not reported (0)
Are exposure data recorded?	Yes (1), no (0)
Is the frequency of data collection reported?	Yes (1), no (0)
If yes:	≥ Daily (3), ≥ weekly (2), ≥ monthly (1), not reported (0)
Is a clear injury definition provided?	Yes (1), no (0)
If yes:	Medical attention (3), time loss (2), other (1), no clear definition (0)
Is the method for assessing exposure described?	Yes (1), no (0)
If yes:	Individual data collection (2), exposure estimated (1), not reported (0)
Is the method for assessing injury reported?	Yes (1), no (0)
If yes:	Briefed medical personnel (3), medical personnel (2), coach, self-report, media reports (1), not reported (0)
Are characteristics of injury reported (location, type, mechanism, severity, recurrent)?	Yes (1), no (0)
If yes:	Complete (2), partly (1), no (0)
Is the drop out < 30% drop out?	Yes (1), no (0)

The identified quality score was used to determine a high (above the median) or low (below the median) study quality of the studies investigated (median score was 18). Two independent reviewers (K.H., A.J.) with a third reviewer (A.L.R.) for consensus assessed the study quality of the included studies.

Publication bias was checked by visual inspection of funnel plots (log risk ratio against standard errors) and regression test for funnel plot asymmetry.

### Data Synthesis and Statistics

To compare injury risk between male and female runners, risk ratios (RR) with 95% confidence intervals (CI) were computed for each study. Meta-analytic pooling was done using a random-effects model (DerSimonian and Laird method [27]). Between-study heterogeneity was estimated using Cochran’s *Q* and *I*^2^ statistics. To reveal potential publication biases, funnel plots were constructed if more than ten studies were available [28]. Besides visually checking their symmetry, Egger’s regression test was applied.

Following the calculation of pooled RRs, we used a mixed-effects meta-regression model to identify variables potentially affecting the outcome of the meta-analysis [27]. The choice of tested moderators (effect modifiers) was based on three criteria: (1) a plausible impact on the tested variables, (2) reporting in the included studies, (3) sufficient variation of the moderators’ values [29]. The following moderators were submitted into the meta-regression model: performance/expertise level (recreational: no competitions, competitive: participating in local competitions, elite: qualifying for national or international competitions); age (youth: < 18, adult: ≥ 18) competition distance (≤ 10 km, > 10 km); study quality (low: study quality score < 18, high: study quality score ≥ 18), training duration (low: < 7.5 h or high: ≥ 7.5 h/week), training mileage (low: < 64 km/week, high: ≥ 64/week). Moderator analyses were performed if ten or more studies were available [28]. If a significant moderator was detected, a subgroup analysis comparing the respective values of the moderator was performed using the meta-analytic procedures described above.

All calculations were performed using algorithms of the metaphor package embedded in R (R Foundations for Statistical Computing, Vienna, Austria) as well as the software JAMOVI [30] and OpenMeta [Analyst] software (OS X version 10.12 obtained from http://www.cebm.brown.edu/openmeta/).

## Results

### Search Results

**Fig. 1:**
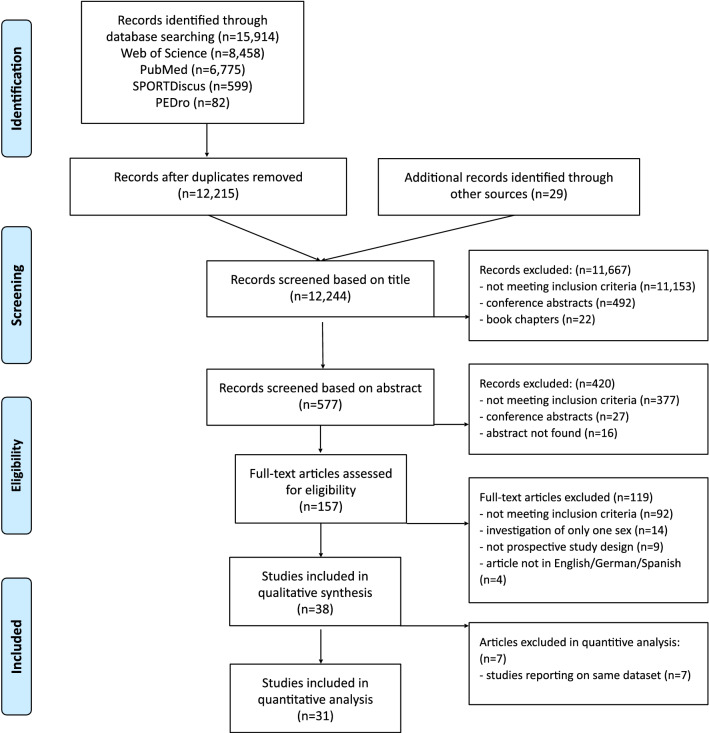
Flow diagram displaying the literature search

The search returned 15,914 studies and 29 additional studies were identified through other sources. After removing 3699 duplicates and applying inclusion criteria, a total of 38 studies were considered eligible [7, 19, 24, 31–65]. Thirty-one of them could be included in the quantitative analysis. Seven studies reported on the same data sets as other included studies and were excluded from the quantitative analysis [19, 31–33, 39, 40, 65]. The full literature search process is displayed in Fig. 1.

### Characteristics of Included Studies

Of the included studies, 36 reported injury data from 35,689 participants (40.8% female). Two studies reporting on injuries from the National Collegiate Athletic Association (NCAA) database did not state the number of athletes but did report the athlete exposure (242,244 athlete exposures with 46.7% females [24] and 276,207 athlete exposures with 50.7% females [59]). Most studies were prospective cohort studies (*n* = 37), while the control group (not receiving any intervention) from one randomised controlled study met the inclusion criteria [42]. Twenty-three studies investigated road runners, 11 track runners (middle and long distance), 10 cross-country runners and 3 studies reported on trail running/orienteering (Table 2). Studies from major competitions (European or World Championships) reported concurrently on track and road running (half or full marathon) [19, 31–33, 41]. Regarding competition level, 18 studies reported on novice and recreational runners, 11 on competitive and 9 on elite runners. Study characteristics of all included studies are summarized in Table 2.

**Table 2:** Study characteristics

Study	Sport	Cohort/populations, (Country)	Level	Number of participants (female/male)	Age (years)	Duration of data collection	Injury definition	Exposure measurement	Injury rates (overall)	Injury rates (female/male)	Risk of bias score
Nicholl et al. [54]	Marathon	Sheffield Marathon (1982) participants	Recreational	53/2236	over 18	1 day	Contact with medical staff at first-aid posts	One full marathon	18 injured runners per 100 marathon runners	Female: 32 injured runners per 100 marathon runners; Male: 17.5 injured runners per 100 marathon runners	14
Hughes et al. [47]	Road racing	Chicago Distance Classic (20 km)	Recreational	188/1071	32.3 (range 9—75)	1 day	Self-reported specific orthopaedic problems	20 km race	28.4 injured runners per 100 runners	Female: 54.3 injured runners per 100 runners; Male: 31.6 injured runners per 100 runners	11
Johansson [49]	Orienteers	College students	Elite	33/56	17.5 ± 1.5	1 year	Time loss training or competition injuries	Daily training logs, monthly reports of training	3 injuries per 1000 h; 74 injuries per 100 runners	Female: 72.7 injuries per 100 runners; Male: 75.0 injuries per 100 runners	20
de Loes and Goldie [38]	Road/Trail	Population based (Sweden)	Recreational	2505/3530	15–59	1 year	Medically diagnosed: injury registry from hospitals and sports medicine physician. Validated by telephone interview	Data were collected from representative sample via questionnaire, then extrapolation to whole population	0.7 injuries per 1000 h	Female: 0.7 injuries per 1000 h; Male: 0.7 injuries per 1000 h	14
McLain and Reynolds [52]	Cross-country	High school students	Competitive (high school)	40/54	NA	1 year	Athletic trainer: Any time loss incident resulting from athletic participation	NA	10.7 injuries per 100 runners	Female: 7 injured runners per 100 runners; Male: 13 injured runners per 100 runners	11
Walter et al. [63]	Road runners (10 Miles)	Community running events (4–22.4 km) in Ontario	Recreational	301/980	over 14	52 weeks	Injuries severe enough to reduce the number of miles run, take medicine, or see a health professional	NA	48.4 injured runners per 100 runners	Female: 45.5 injured runners per 100 runners; Male: 49.3 injured runners per 100 runners	13
Bennell et al. [35]	Track	Victoria athletics	Competitive (college)	26/28	17–26	48 weeks	Stress fracture: medical imaging after clinical evaluation	Structured interview: hours per week, weeks without running,	0.7 stress fractures per 1000 h 25.9 runners with stress fractures per 100 runners	Female: 30.8 runners with stress fractures per 100 runners; Male: 21.4 runners with stress fractures per 100 runners	21
Beachy et al. [34]	Cross-country	High school students (Punahou, Hawai)	Competitive (high school)	787/501	NA	8 years	Any athlete complaint that required the attention of the athletic trainer, regardless of the time lost from activity	NA	65 injuries per 100 runners	Female: 65 injuries per 100 girls; Male: 66 injuries per 100 boys	14
Colbert et al. [36]	Road running	Patients from Cooper Clinic	Recreational	220/1771	NA	8 years	Clinical visit	NA	26.3 injured runners per 100 runners	Female: 25.0 injured runners per 100 runners; Male: 26.4 injured runners per 100 runners	9
Rauh et al. [58]	Cross country	High school students (Washington state)	Competitive (high school)	1202/2031	NA	15 years	An injury was defined as a medical problem resulting from athletic participation that required an athlete to be removed from a practice or competitive event or to miss a subsequent practice or competitive event	An AE was defined as any practice or meet (competition) in which there was the possibility of sustaining an athletic injury	13.1 injuries per 1000 AEs	Female: 16.7 injuries per 1000 AEs; Male: 10.9 injuries per 1000 AEs	18
Steinacker et al. [61]	Marathon	Berlin Marathon participants	Recreational	22/36	44.5	24 weeks	Self-reported orthopaedic problems (Survey)	NA	46.6 injured runners per 100 runner	Female: 41.6 injured runners per 100 runners; Male: 54.5 injured runners per 100 runners	11
Taunton et al. [62]	Road race (10 km)	Vancouver Sun Run (10 km)	Recreational	635/205	NA	13 weeks	Self-reported pain (Survey) with medical confirmation	NA	29.5 injured runners per 100 runner	Female: 30.2 injured runners per 100 runners; Male: 28.3 injured runners per 100 runners	14
Dane et al. [37]	Road running	College students	Competitive (college)	47/45	17–28	One season (12 weeks)	Medically diagnosed: contusions, bleeding, wounds, and fractures, except small bruises, were classified as injuries	NA	57.1 injured runners per 100 runners	Female: 52 injured runners per 100 runners; Male: 60 injured runners per 100 runners	10
Rauh et al. [57]	Cross country	College students (Seattle)	Competitive (college)	186/235	NA	One season	Muscle, joint, or bone problem/injuries of the back or lower extremity requiring the runner to be removed from a practice or meet or to miss a subsequent one	An AE was any practice or competitive event where a runner was at risk of sustaining an injury	17.0 injuries per 1000 AEs	Female: 19.6 injuries per 1000 AEs; Male: 15.0 injuries per 1000 AEs	20
Plisky et al. [56]	Cross country	High school students (Wisconsin)	Competitive (college)	46/59	NA	13 weeks	Medial tibial stress fracture: pain in the tibial region, exacerbated with repetitive weight-bearing activity, and localized pain with pal- pation along the distal two thirds of the posterior-medial tibia	AE: any practice or competitive event	2.8 stress fractures per 1000 AEs	Female: 4.3 stress fractures per 1000 AEs; Male: 1.7 stress fractures per 1000 AEs	21
Alonso et al. [32]	Track + Marathon	2007 IAAF World championships (Osaka) participants	Elite	249/267	17–37	9 days	All musculoskeletal injuries regardless of the consequences with respect to the athlete’s absence from competition or training	Number of competing athletes	150 competition injuries per 1000 athletes	Time-loss injuries per 1000 registered athletes - Female: 800 m: 22, 1500 m: 26, 3000 m SC: 48, 5000 m: 38, 10000 m: 158, marathon 61; - Male: 800 m: 43, 1500 m: 24, 3000 m SC: 79, 10000 m: 91, marathon: 118	22
Alonso et al. [33]	Track + Marathon	2009 IAAF World championships (Berlin) participants	Elite	244/312	NA	9 days	All time-loss musculoskeletal injuries (traumatic and overuse) regardless of the consequences with respect to the athlete’s absence from competition or training	The number of competing athletes was defined as all athletes who started at least once in a discipline	MD: 173.3 injuries per 1000 registered athlete LD: 151.1 injuries per 1000 registered athletes	Injuries per 1000 registered athletes - Female: 800 m 46.5 1500 m 71.4 3000 m SC: 48.8 5000 m 43.5 10000 m 90.9 Marathon 0 - Male: 800 m 0 1500 m 37.0 3000 m SC: 26.5 5000 m 102.6 10000 m 32.3 Marathon 30.6	22
Buist et al. [7]	Road racing (4 Miles)	Groningen 4 mile	Recreational	422/207	43.7 ± 9.5	8 weeks	Any time loss running-related musculoskeletal pain at the lower extremity or back	Exposure as given by training programme	25.9 injured runner per 100 runners; 30.1 injuries per 1000 h	Injury incidence rate per 1000 h - Female: 27.5, Male: 35.0 Mean Prevalence - Female: 23.4 injured runners per 100 runners Male: 31.4 injured runners per 100 runners	18
Alonso et al. [31]	Track + Marathon	2011 IAAF World championship (Daegue) participants	Elite	208/268	17–42	9 days	All musculoskeletal injuries regardless of the consequences with respect to the athlete’s absence from competition or training	Number of competing athletes	MD: 176.1 injuries per 1000 registered athletes LD: 187.8 injuries per 1000 registered athletes	Time-loss injuries per 1000 registered athletes - Female: 800 m: 55.6, 1500 m: 57.1, 3000 m SC: 0, 5000 m: 125 injuries, 10000 m: 52.6,, marathon 53.6; - Male: 800 m: 22.7, 1500 m: 76.9, 3000 m SC: 0, 5000 m: 122 injuries, 10000 m: 47.6 injuries marathon: 220.6	22
Jacobsson et al. [48]	Track (MD + LD)	Swedish national team	Elite	54/55	17–37	52 weeks	Self-reported musculoskeletal pain, soreness or injury with time loss or changes in normal training/competition	Self-reported athletic training participation	83 injured runners per 100 runners	Adults: Female: 74 injured runners per 100 runners; Male: 81 injured runners per 100 runners Youth: Female: 57 injured runners per 100 runners; Male: 58 injured runners per 100 runners	19
Edouard et al. [40]	Track (MD)	European Athletics indoor championships Paris 2011 participants	Elite	125/75	NA	3 days	Any musculoskeletal complaint and concussion that received medical attention regardless of time loss	Athletes’ exposure in competition	MD: 53 injuries per 1000 registered athletes	Injuries per 1000 registered athletes – Female: 800 m: 47.6 3000 m 150.0 - Male: 800 m: 107.1 3000 m 34.5	21
Nielsen et al. [55]	Road racing	DANO-RUN study	Novice runners	441/432	37.2 ± 10.3	1 year	Any musculoskeletal complaint of the lower extremity or back caused by running that restricted the amount of running for at least 1 week	Online diary: GPS or manually kilometers	23.1 injured runner per 100 runners	Female: 21.8 injured runners per 100 runners; Male: 24.5 injured runners per 100 runners	23
Edouard et al. [39]	Track (MD)	European Athletics championships Helsinki 2012 participants	Elite	66/164	NA	3 days	Any musculoskeletal complaint and concussion that received medical attention regardless of time loss	Athletes’ exposure in competition	MD: 53 injuries per 1000 registered athletes	Injuries per 1000 registered athletes -Female: 800 m: 41.7 1500 m: 30.3 3000 m: 142.9 5000 m: 347.8 10000 m: 176.5 - Male: 800 m: 69.8, 1500 m: 171.4, 3000 m: 275.9 5000 m: 71.4 10000 m 103.4	21
Changstrom et al. [24]	Cross-country	National High School Sports- Related Injury Surveillance System (2011–2012), (USA)	Competitive (high school)	NA	13–19	2 years	(Stress) fractures, concussions and dental injuries with or without time loss. All injuries with time loss requiring medical attention	Athlete exposure (AE)	7.8 stress fractures per 100,000 AEs	Female: 10.6 stress fractures per 100,000 AEs; Male: 5.4 stress fractures per 100,000 AEs	19
Edouard et al. [19]	Track + Marathon	All athletic world championships (2007–2014)	Elite	1302/1573	NA	(3–9 days per championship)	All musculoskeletal injuries (traumatic and overuse) and concussion newly incurred during competition or training regardless of the consequences with respect to the athlete’s absence from competition or training	Total number of registered athletes	N/A	Injuries per 1000 registered athletes - Female: MD 94.6 LD 155.3 Marathon 153.3 - Male: MD 108.5 LD 141.4 Marathon 195.5	21
Kluitenberg et al. [50]	Road racing	NLstart2run	Novice runners	1332/364	43.3	6 weeks	A musculoskeletal complaint of the lower extremity or back that hampered running ability for three consecutive training sessions	Weekly running frequency and running exposure (in minutes) for each training session	27.5 injuries per 1000 h	Female: 10.4 injured runners per 100 runners; Male: 12.6 injured runners per 100 runners	17
Hespanhol Junior et al. [45]	Road racing (10 Miles)	Tilburg Ten Miles	Recreational	31/22	44.1	18 weeks	OSTRC: All running-related injuries	Online questionnaires completed every fortnight	Mean prevalence: 30.8 injured runners per 100 runners and cumulative prevalence 60.4 injured runners per 100 runners	Mean prevalence Female: 11.5% injured runners per 100 runners Male: 19.3 injured runners per 100 runners	18
Hespanhol Junior et al. [44]	Trailrunning	Dutch trail runners	Recreational	57/171	43.4	6 months	OSTRC: All running-related injuries	Online questionnaires completed every fortnight	10.7 injuries per 1000 h; mean prevalence 22.4%	Injury incidence rate per 1000 h - Female 9.1 Male: 11.3 Mean Prevalence: Female: 20.7%; Male: 23.0%	17
Rizzone et al. [59]	Cross country	NCAA (2004–2014)	Competitive (college)	NA	NA	9 years	Time loss stress fractures that required medical attention: (1) occurred due to participation in a school-sanctioned practice or competition, (2) required attention from an AT or physician, (3) resulted in at least 24 h of time missed from participation, and (4) had a reported diagnosis of stress fracture	AE: 1 student-athlete participating in 1 NCAA-sanctioned practice or competition	22.4 stress fractures per 100,000 AEs	Female: 28.6 stress fractures per 100,000 AEs; Male: 16.1 stress fractures per 100,000 AEs	18
Messier et al. [53]	Road racing	TRAILS study	Recreational	128/172	Range 18—60	104 weeks	Overuse running injuries: grade 1, maintained full activity in spite of symptoms; grade 2, reduced weekly mileage; and grade 3, interrupted all training for at least 2 weeks	NA	66 injured runners per 100 runners	Female: 73 injured runners per 100 runners; Male: 62 injured runners per 100 runners	15
Winter et al. [64]	Road running	Runners from local running club	Recreational + Competitive	35/57	18—65	52 weeks	Pain preventing the runner from performing or completing at least one training session	Training diary with information on running sessions per week, distance and duration of runs	51.3 injured runners per 100 runners	Female: 54.8 injured runners per 100 runners; Male: 48.9 injured runners per 100 runners	20
Fokkema et al. [42]	Road racing	INSPIRE trial (NN City Pier City The Hague, NN Marathon Rotterdam, Ladies Run Rotterdam)	Recreational	553/629 (Control group)	41.4 ± 12	4.5 ± 1.6 months	Injuries of the lower back or lower extremities caused by running with change of training for at least 1 week, a medical visit or medication	NA	36.7 injured runners per 100 runners	Female: 35.8 injured runners per 100 runners; Male: 38.3 injured runners per 100 runners	15
Hayes et al. [43]	Cross-country	NCAA (Ivy League & New England Small College Athletics)	Competitive (college)	57/10	17–21	1 US-cross-country season	Self-reported Injuries that were not present during administration of the pre-season survey	Average and maximum weekly mileage in increments of 10 miles (e.g. 31–40 miles per week)	53 injured runners per 100 runners (over one season)	Female: 51 injured runners per 100 runners; Male: 55 injured runners per 100 runners	12
Lagas et al. [51]	Road racing	INSPIRE trial (NN City Pier City The Hague, NN Marathon Rotterdam, Ladies Run Rotterdam)	Recreational	909/1020	41.9 ± 12.1	20.5 ± 7 weeks	Self-reported Achilles tendinopathy caused by running with change of training for at least 1 week, a medical visit or medication	NA	5.2 injured runners per 100 runners	Female: 3.6 injured runners per 100 runners; Male: 6.6 injured runners per 100 runners	12
Ruffe et al. [60]	Cross-country	High school students (California)	Competitive (high school)	80/68	15.6	1 US-cross-country season	Muscle, bone, or joint problem/injury of the low back or lower extremity requiring removal from training/competitions or leading to missed subsequent training/ competitions	Runners’ daily participation in practices and competitive events	33.1 injured runners per 100 runners (over one season)	Female: 38.8 injured runners per 100 runners; Male: 26.5 injured runners per 100 runners	16
Winter et al. [65]	Road running	Runners from local running club	Recreational + Competitive	35/57	18—65	52 weeks	Pain preventing the runner from performing or completing at least one training session assessed by an experienced health or medical professional	Average kilometers per week; average duration (minutes) per week; average frequency per week	51.3 injured runners per 100 runners	Female: 54.8 injured runners per 100 runners; Male: 48.9 injured runners per 100 runners	21
Edouard et al. [41]	Track + Marathon	IAAF World and European Championships participants	Elite	MD: 742/943; LD 656/793; Marathon 464/550	NA	78 days (3–9 days per championship)	All musculoskeletal injuries (traumatic and overuse) and concussion newly incurred during competition or training regardless of the consequences with respect to the athlete’s absence from competition or training	Total number of registered athletes	Injuries per 1000 registered athletes—MD: 97 LD: 126 Marathon: 139	Injuries per 1000 registered athletes - Female: MD 84.9 LD 128 Marathon 118.5 Male: MD 106 LD 123.6 Marathon 156.4	19
Hofstede et al. [46]	Half- + Marathon	SUMMUM study (Utrecht Marathon)	Recreational	71/90	40.7 ± 11.7	16 weeks	OSTRC questionnaire, injuries with a moderate to severe reduction in training or competition or time loss	NA	44.1 substantial injuries per 100 runners	Female: 52.1 substantial injuries per 100 runners; Male: 37.8 substantial injuries per 100 runners	14

*NA* not available, *3000 m SC* 3000 m steeplechase, *AE* athlete exposure, *MD* middle distance, *LD* long distance, *IAAF* International Amateur Athletics Federation, *GPS* global positioning system, *NCAA* National Collegiate Athletic Association, *OSTRC* Oslo Sports Trauma Research Center

### Study Quality

The two independent reviewers evaluating study quality agreed on 441 of 570 evaluated items (agreement = 77.4%). The scores for study quality ranged between 9 and 23 out of 24 points with a median of 18 and a mean ± SD of 16.8 ± 4.1. Most studies (> 90%) reported recruitment procedures, injury assessment and documented injury characteristics. Fewer studies achieved maximal points due to not recording individual exposure data (50.0% of maximal points) or exposure data at all (57.9% of maximal points) as well as not using a medical attention definition (56.1% of maximal points). The results of the study quality assessment are presented in Electronic Supplementary Material Table S1.

**Fig. 2:**
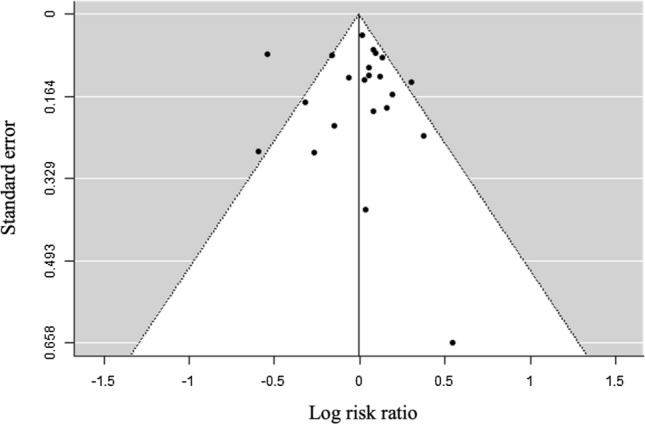
Funnel plot of the overall differences between injury rate of female and male runners (log risk ratios against standard error)

Except for one outlier [47], visual inspection of the funnel plot (Fig. 2) showed a symmetrical distribution of the log risk ratios and the regression test for funnel plot asymmetry (− 0.150; *p*= 0.881) suggested no indication of publication bias.

### Overall Injury Rates

**Fig. 3:**
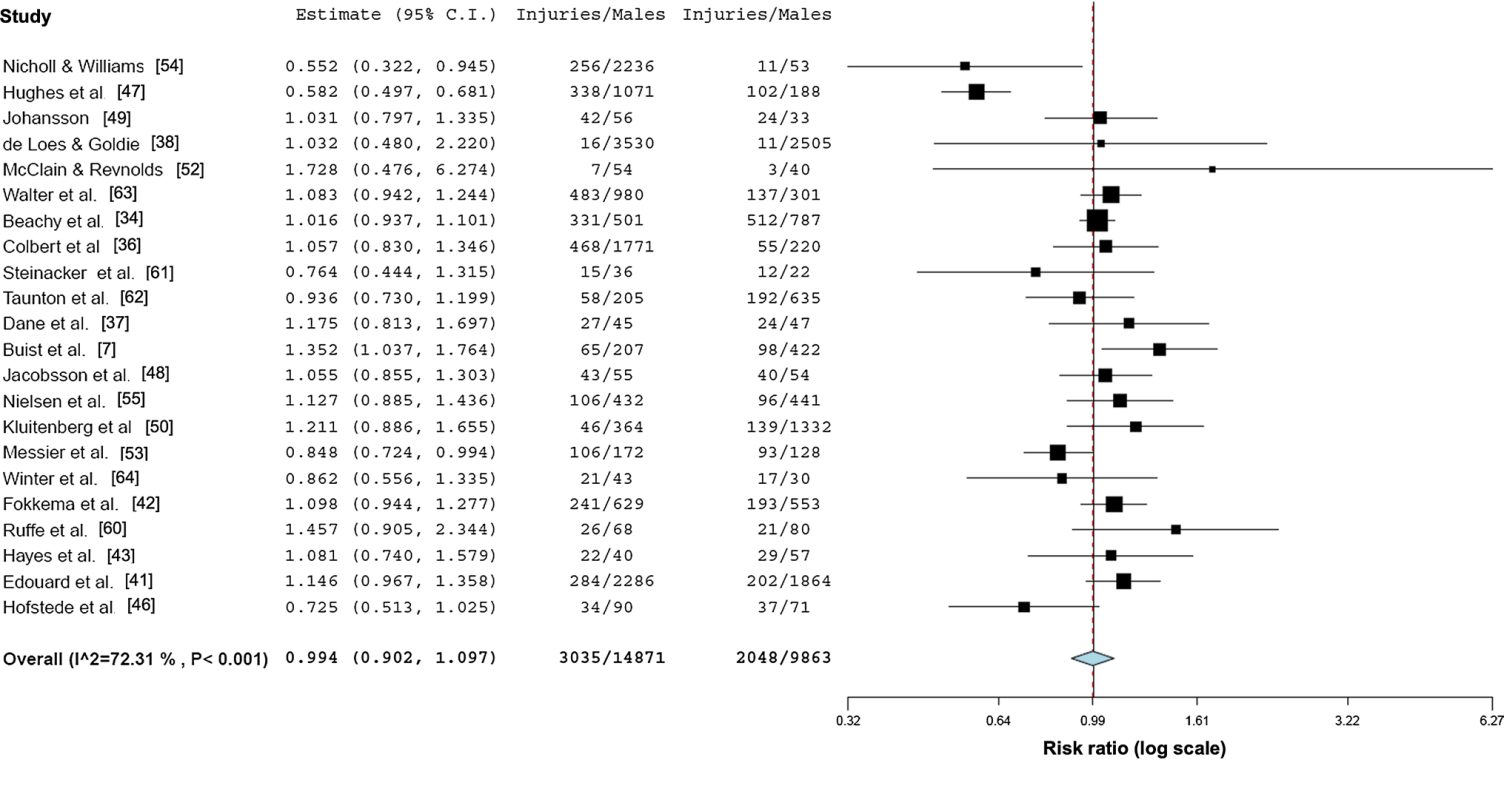
Forest plot depicting the meta-analytical results comparing risk ratios for male and female runners regarding injuries per 100 runners

**Fig. 4:**
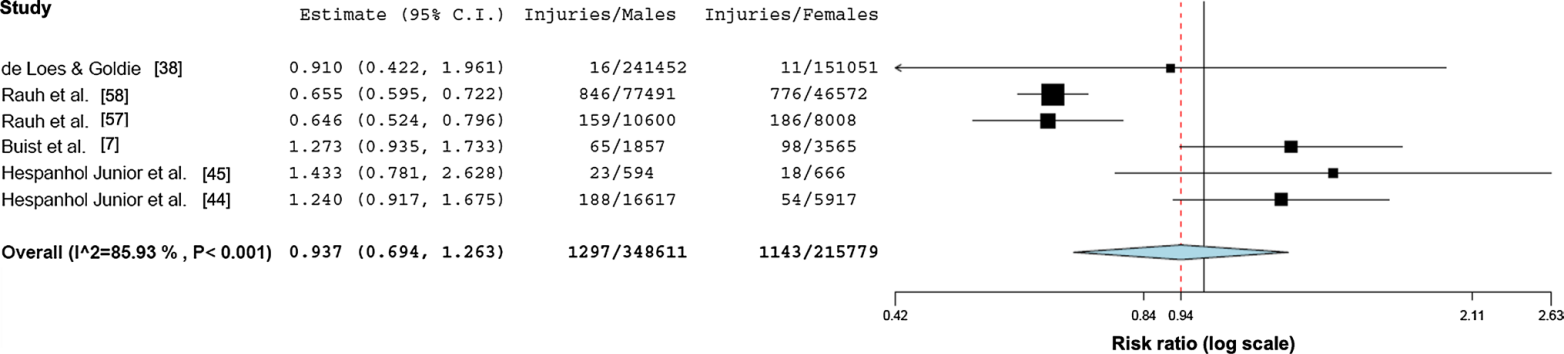
Forest plot depicting the meta-analytical results comparing risk ratios for male and female runners regarding injuries per exposure (hours or athlete exposures)

The overall injury rate was 20.4 (95% CI 19.7–21.1) injuries per 100 male runners and 20.8 (95% CI 19.9–21.7) injuries per 100 female runners. Meta-analytic pooling did not reveal differences between female and male runners’ injury rates per runner (*n* = 21; RR 0.99, 95% CI 0.90–1.10; *p* = 0.84; I^2^ = 72.31) or per specific exposures (*n* = 6; RR 0.94, 95% CI 0.69–1.27;  *p* = 0.669; *I*^2^ = 85.93) (Figs. 3 and 4). Due to the small number (*n* = 6) of studies reporting injuries per exposure (athlete exposures (*n* = 2) or hours (*n* = 4)), no aggregation of overall injury rates per specific exposures was performed.

### Meta-Regression

**Fig. 5:**
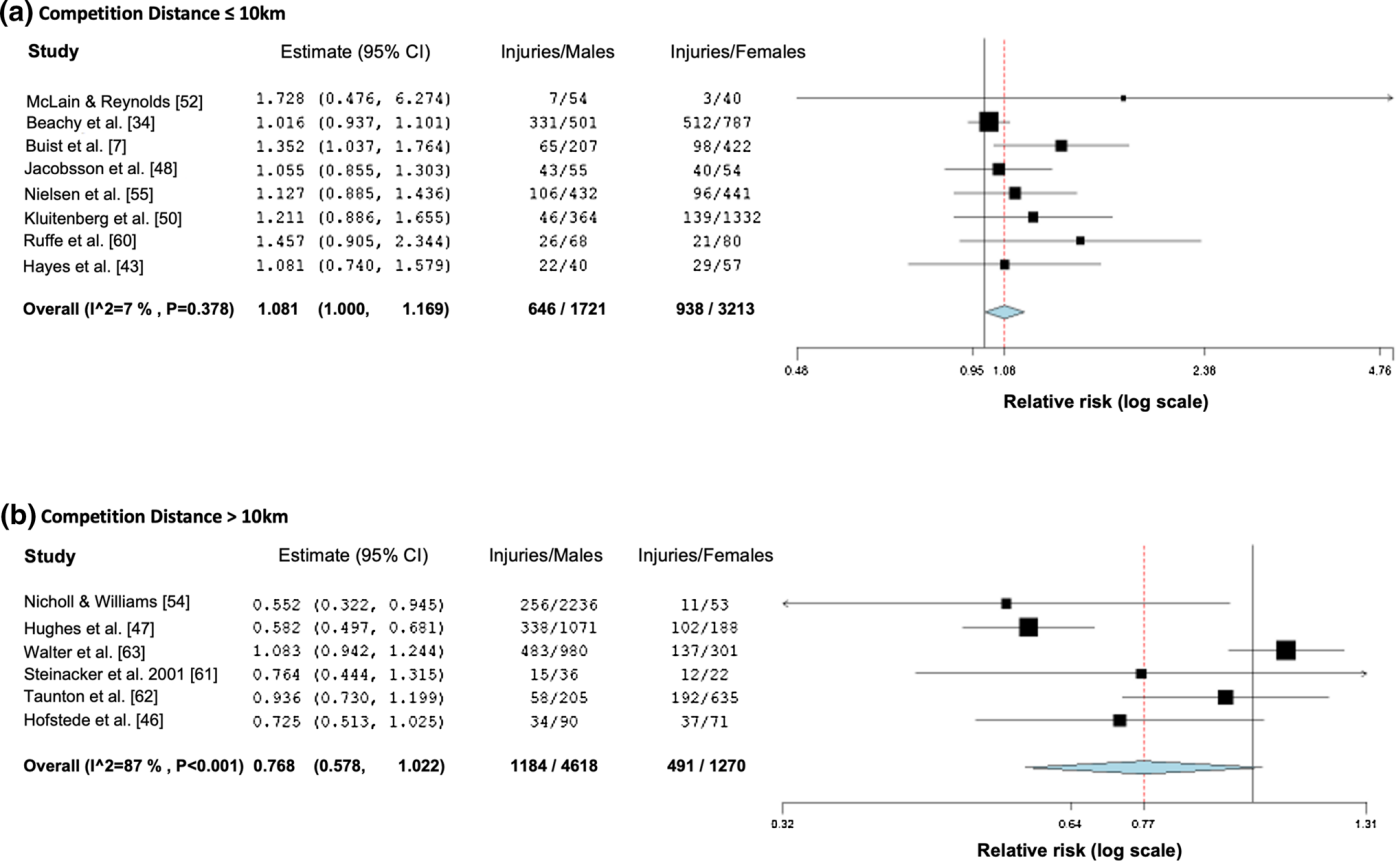
Forest plot depicting the meta-analytical results for sub-analysis (competition distance) of risk ratios for male and female runners regarding injuries per 100 runners. Subgroup 1 (**a**) represents studies investigating runners competing in distances below or equal to 10 km and subgroup 2 (**b**) in distances above 10 km

Moderator analyses of injury RR rates per runner revealed an association of a higher injury risk in men and competition distances exceeding distances of 10 km (*p* = 0.002) (Table 3). Specifically, the subgroup meta-analysis of competition distance showed a significantly higher RR of 1.08 (95% CI: 1.04–1.39) for female runners with competition distances ≤ 10 km. For competition distances > 10 km, the comparison approached but failed to reach significance although the RR of 0.77 (95% CI: 0.58–1.02) was suggestive of a lower probability of injury in male runners (Fig. 5). No meta-regression was performed for specific injuries and moderators training duration or training mileage due to absence of more than ten studies reporting these variables [28].

**Table 3:** Results of the moderator analysis for injury risk ratio rates per 100 female or male runners

Moderator	No of comparisons (k)	*Z*	*p*	Risk ratio estimate (95% CI)	Tau^2^/*Q*
**Risk of bias**	22				0.0299/68.1
Intercept		− 0.88	0.378	− 0.051 (− 0.167 to 0.063)	
Moderator		1.42	0.156	0.151 (− 0.058 to 0.359)	
**Level**	20				0.0385/63.1
Intercept		0.68	0.495	0.044 (− 0.083 to 0.171)	
Moderator		1.05	0.293	0.110 (− 0.095 to 0.316)	
**Age**	15				0.0224/27.3
Intercept		0.87	0.387	0.116 (− 0.146 to 0.378)	
Moderator		− 0.81	0.419	− 0.119 (− 0.407 to 0.170)	
**Competition distance**	14				0.0311/44.7
Intercept		1.71	0.088	0.144 (− 0.021 to 0.309)	
Moderator		− 3.05	0.002	− 0.387 (− 0.636 to − 0.138)	

*95% CI* 95% confidence interval

### Specific Injury Rates

Data for two specific running-related injuries were available for synthesis.

#### Bone Stress Injuries

**Fig. 6:**
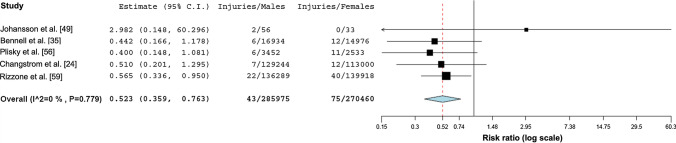
Forest plot depicting the meta-analytical results comparing risk ratios for male and female runners regarding bone stress injuries

Four studies reported on bone stress injuries with a pooled decreased probability for male runners (estimated RR 0.52, 95% CI 0.36–0.76, * p* < 0.001; * I*^2^ = 0) (Fig. 6).

#### Achilles Tendinopathy

**Fig. 7:**
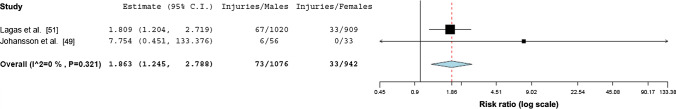
Forest plot depicting the meta-analytical results comparing risk ratios for male and female runners regarding Achilles tendinopathy

Furthermore, data pooling for two studies reporting injury rates for Achilles tendinopathy revealed an increased chance for male runners to have an Achilles tendon injury (estimated RR 1.86, 95% CI 1.25–2.79, *p* = 0.022; * I*^2^ = 0%) (Fig. 7).

## Discussion

The aim of this analysis was to systematically analyse the literature to reveal sex-related differences in running-related injury rates and characteristics. While no differences between sexes were found for overall running-related injuries, female runners were more likely to sustain bone stress injuries while male runner were more prone to Achilles tendinopathies. Meta-regression showed that for competition distances of 10 km and shorter, female runners had higher risk for injuries than male runners.

### No Differences in Overall Injury Rates between Female and Male Runners

Despite pooling data from all available epidemiological studies, no differences in overall injury rates between female and male runners were found in this systematic review. This was the case for both studies reporting injuries per runner and injuries per specific exposures. The injury rates of 20.4 (male) and 20.8 (female) per 100 runners are in accordance with summaries of injury rates from the last three decades [4]. Nonetheless, these rates are at the lower spectrum of published injury rates that were reported to be up to 79.3% [66].

### Shorter Competition Distances Increase the Risk of Injury for Female Runners

Injury rates depend on several factors that need to be taken into consideration, such as systematic factors (age, BMI), running-/training-related factors (training frequency, training and racing distance, experience, level of running, footwear, biomechanics), health factors (injury history) and lifestyle factors (drinking, smoking) [66–70].

Not all of these factors were reported in each study and may vary between investigated populations. Therefore, the moderator analysis was incorporated into this study. Only competition distance was a statistically significant moderator for an increased risk of female runners compared to male runners when running competition distances of 10 km and shorter. Furthermore, the subanalysis revealed a tendency of increased injury risk for male runners for longer distances than 10 km. This is in accordance with the finding that male runners had a higher risk of sustaining injuries compared to female runners when running high mileages (> 64 km/week) [18]. While running higher mileages are associated with longer competition distances, this can only be used as an estimate for this discussion. Unfortunately, there was insufficient reporting of training load (time or mileage) in the included studies. For future studies reporting data on injury epidemiology or risk factors, it is strongly recommended to report the training load [71, 72].

### Bone Stress Injuries Occur Twice as Often in Female than in Male Runners

Female runners had twofold higher risk of having a bone stress injury compared to male runners in this review. A bone stress injury is an injury pattern with known sex differences for epidemiology and risk factors [73]. Bone stress injuries are common running-related overuse injuries due to cumulative microtrauma to the bone [74]. Especially in younger ages, females seem to have a higher risk for bone stress injuries compared to male runners. For example, Changstrom et al. [24] reported a twofold risk and Plisky et al. [56] a 2.5-fold risk for female high school runners of sustaining a bone stress injury compared to male high school runners in cross-country. In older collegiate athletes, female cross-country runners were found to have 28.6 injuries per 100,000 athlete exposures (AE) compared to 16.4 injuries per 100,000 AE in males, representing a statistically significant rate ratio of 1.8 [59]. In outdoor track (100 m–1500 m), this difference was even higher (22.3 injuries/100,000AE for females and 7.2 injuries/100,000AE for males, risk ratio of 3.1) [59]. One possible explanation that has been discussed was the association of bone stress injuries with the female athlete triad (low energy availability, menstrual disturbance and low bone mineral density) to explain the higher risk for bone stress injuries in female runners [35, 75, 76]. However, while the term female athlete triad is used only for female athletes, the more current and more detailed concept of relative energy deficiency of sports (RED-S) has also been described for male athletes [77–79]. Despite using the same initial treatment (activity modification, protected or non-weight bearing) of bone stress injuries for both sexes, the further treatment differs between female and male runners, depending on specific risk factors, such as elevated RED-S risk, biomechanics (load rates, hip adduction, rearfoot eversion), altered hormonal status or calcium and vitamin D intake [14, 15, 73, 80]. In summary, bone stress injuries are more prevalent in female runners and treatment/rehabilitation strategies should incorporate sex as an important variable. Nonetheless, in the prevention of bone stress injuries consideration of the sex would probably benefit from awareness of RED-S, including screening for low energy availability and low mineral bone density.

### Achilles Tendinopathies Occur Twice as Often in Male Compared to Female Runners

Data from two studies showed that male runners had almost twice the risk of having an Achilles tendinopathy as female runners [49, 51]. This is in accordance with a systematic review on the pathogenesis of Achilles tendinopathy [81]. The Achilles tendon transmits the generated forces from the gastrocnemius-soleus muscle complex and, thus, is an important tendon for propulsion during running. However, the Achilles tendon has a poor blood supply and, therefore, is prone to overuse injuries, such as a tendinopathy [81]. The lifetime prevalence has been reported as high as 40–50% in runners [13, 82] and a recent 1-year prospective study determined the incidence rate in a cohort of recreational runners to be 5.2% [51]. While the amount of loading is the key factor in the etiology of Achilles tendinopathy, there are several intrinsic (age, stress, genes, biomechanics, body composition) and extrinsic factors (footwear) modulating the risk for this injury [83]. Recent studies found biomechanical (footstrike pattern, ankle dorsiflexion moments) and training-related parameters (changes in training, cold weather, footwear, use of compression socks, mileage) as possible risk factors [10, 51, 84–86]. This summary of (possible) risk factors does not directly explain the increased probability for male runners to have an Achilles tendinopathy. Therefore, we can only speculate about the possible mechanisms. One recently published study discusses the mechanism of the lifetime cumulated load (together with running years) which might be higher in male runners than in female runners [87]. Chronic loading needs to be taken into account when evaluating the risk for Achilles tendinopathies.

Another explanation might be found in the hormonal differences between women and men. For example, estrogen is associated with collagen synthesis and could therefore influence tendon healing capacity [88, 89]. Furthermore, estrogen deficiency has been reported to negatively affect tendon metabolism and healing [90]. Hormonal fluctuations that are typical for the menstrual cycle have not been associated with modifications of tendon function [90, 91]. A review summarizes that high or low levels of sexual hormones (estrogen, progesterone and testosterone) are not directly causing tendinopathies but may play a role in tendon pathologies [92]. Therefore, individual hormonal status should be taken into account for injury risk of female and male runners as well as for their therapies and prevention [92].

### Results of the Current Review in Contrast with and in Addition to Other Systematic Reviews

This was the first systematic review on sex-specific differences in running injuries incorporating a meta-regression analysis to determine moderating variables and shall be discussed in light of other systematic reviews on this topic.

This systematic review contrasts the findings of the systematic review by van der Worp et al. [18], who found female runners at a lower overall risk of sustaining an injury than male runners. This finding was particularly found in men under 40 years. However, when assessing the evidence level the authors called for caution in the interpretation of their findings since these were based on only five high-quality and one low-quality studies. In contrast, our review included epidemiological studies reporting injury rates separately for both sexes. With this approach, 26 studies were included and meta-analyses showed no sex differences for overall running injuries when calculated per runner or per exposure (hours or AE). Furthermore, we were able to conduct a meta-regression analysis showing a higher injury risk for female runners in competition distances of 10 km and shorter as well as a tendency for a higher injury risk for male runners in competition distances longer than 10 km. This is a new finding and in line with the increased risk for male runners with a high weekly mileage (> 64 km), which is typically needed for longer competition distances [18].

The systematic review by Wright et al. [93] found female sex to be a primary risk factor for lower extremity bone stress injuries despite conflicting evidence using an exploratory meta-analysis incorporating three etiological studies [6, 94, 95]. The meta-analysis found a similar 2.3-fold increased probability for female runners. Our meta-analysis supports these findings and underlines the evidence for female runners to be more prone to bone stress injuries based on five included prospective studies [24, 35, 49, 56, 59]. Female sex as a risk factor for medial tibial stress syndrome has also been described by a meta-analysis in active individuals (not exclusively runners [96]).

### Limitations and Methodological Considerations of Current Research

This systematic review summarised data from 38 prospective studies representing more than 35,689 participants (from 36 studies) and 518,000 athlete exposures (from 2 studies). While the distribution between female and male runners (40.8–50.7% females) was similar and no overall differences were found, breakdown of injury data regarding sex and according to location or diagnosis was only possible in six studies. Consequently, the available literature included in this systematic review did not allow conclusions on the sex-dependent epidemiology of pathologies other than bone stress injuries and Achilles tendinopathies.

The meta-regression approach of this study included several potential moderators. However, considering the multifactorial aetiology of running-related injuries, other confounding bias such as biomechanical or psychological variables may have influenced the injury risk. Another limitation was the moderate to high heterogeneity of studies included in the overall injury meta-analyses, emphasizing the need for further studies with a clear injury definition and uniform data collection methods [71, 97].

Regarding quality, future studies would benefit from documenting exposure data and using medical attention injury definitions. Furthermore, moderator analysis was only possible for 1 outcome (overall injuries per 100 runners) due to missing descriptive information on study populations (such as mileage, training duration, competition distances). As seen in Table 2, there are several different data collection methods applied and injury definitions used to determine a running injury. In accordance with recent consensus statements in injury epidemiology [71] and a Delphi consensus on running injuries [98], we encourage future running injury research to follow these guidelines to improve the homogeneity of studies. From this, future meta-analyses would benefit from comparing rates of injuries between studies [97, 99].

## Conclusion

Sex does not seem to represent a specific risk factor when considering the overall occurrence of injuries in running. However, female runners more frequently sustain bone stress injuries, while male runners have higher risk of developing Achilles tendinopathies. Preventive measures targeting these diagnoses may therefore be more effective when accounting for sex-specific aspects such as hormonal changes or biomechanical characteristics. Regarding moderators, there is a paucity of evidence although meta-regression identified running competition distance (cut-off 10 km) as a factor associated with higher injury rates in male runners.

## Supplementary Information

Below is the link to the electronic supplementary material.

**Electronic Supplementary Material Table S1**. Study quality results. (DOCX 38 KB)

## Declarations

### Data Availability Statement

The data from the current study are presented in the article/electronic supplementary material and are available from the corresponding author upon request.
