# Supplementary material for: Sex-Specific Differences in Running Injuries: A Systematic Review with Meta-Analysis and Meta-Regression
Source: Sports Med. 2021 Jan 12;51(5):1011–39. doi: 10.1007/s40279-020-01412-7 (PMC8053184; doi:10.1007/s40279-020-01412-7)
Supplement: Supplementary file 1 — Electronic Supplementary Material Table S1. Study quality results. (DOCX 38 KB) [file 40279_2020_1412_MOESM1_ESM.docx]

| **Study** | **Year** | **Are the sources and methods of participant recruitment clearly described? Yes (1), no (0)** | **Are the relevant characteristics (n, age, sex, sport, level of competition) of the study population reported? Yes (1), no (0)** | **Does the study cover season and/or tournaments/championship? Season (2) Tournaments (1), not reported (0)** | **Are exposure data recorded? Yes (1), no (0)** | **Is the frequency of data collection reported? Yes (1), no (0)** | **If yes,: ≥daily (3), ≥weekly (2), ≥monthly (1), not reported (0) (bei Mehrfahnennungen immer die höchste Frequenz)** | **Is a clear injury definition provided? Yes (1), no (0)** | **If yes, medical attention (3), time loss (2), other (1), no clear definition (0)** | **Is the method for assessing exposure described? Yes (1), no (0)** | **If yes , individual data collection (2), exposure estimated (1), not reported (0)** | **Is the method for assessing injury reported? Yes (1), no (0)** | **If yes briefed medical personal (3), medical personal (2), coach, self-report, media reports (1), not reported (0)** | **Are Characteristics of injury reported (location, type, mechanism, severity, recurrent)? Yes (1), no (0)** | **If yes, complete (2), partly (1), no (0)** | **Is the drop out <30% drop out? Yes (1), no (0)** | **Sum** |
| --- | --- | --- | --- | --- | --- | --- | --- | --- | --- | --- | --- | --- | --- | --- | --- | --- | --- |
| Nicholl et al. [54] | 1983 | 1 | 1 | 1 | 0 | 1 | 3 | 0 | 0 | 0 | 0 | 1 | 3 | 1 | 1 | 1 | **14** |
| Hughes et al. [47] | 1985 | 1 | 1 | 1 | 0 | 1 | 3 | 0 | 0 | 0 | 0 | 1 | 1 | 1 | 1 | 0 | **11** |
| Johansson [49] | 1986 | 1 | 1 | 2 | 0 | 1 | 3 | 1 | 2 | 1 | 2 | 1 | 1 | 1 | 2 | 1 | **20** |
| de Loes et al. [38] | 1988 | 1 | 1 | 2 | 1 | 0 | 0 | 0 | 0 | 1 | 1 | 1 | 2 | 1 | 2 | 1 | **14** |
| McLain et al. [52] | 1989 | 1 | 1 | 2 | 0 | 0 | 0 | 1 | 2 | 0 | 0 | 1 | 1 | 1 | 1 | 0 | **11** |
| Walter et al. [63] | 1989 | 1 | 1 | 2 | 0 | 1 | 1 | 1 | 1 | 0 | 0 | 1 | 1 | 1 | 1 | 1 | **13** |
| Bennell et al. [35] | 1996 | 1 | 1 | 2 | 1 | 1 | 1 | 1 | 3 | 1 | 2 | 1 | 3 | 1 | 1 | 1 | **21** |
| Beachy et al. [34] | 1997 | 1 | 1 | 2 | 0 | 0 | 0 | 1 | 3 | 0 | 0 | 1 | 3 | 1 | 1 | 0 | **14** |
| Colbert et al. [36] | 2000 | 1 | 1 | 2 | 0 | 0 | 0 | 0 | 0 | 1 | 1 | 1 | 1 | 0 | 0 | 1 | **9** |
| Rauh et al. [58] | 2000 | 1 | 0 | 2 | 1 | 1 | 3 | 1 | 2 | 1 | 2 | 1 | 1 | 1 | 1 | 0 | **18** |
| Steinacker et al. [61] | 2001 | 1 | 1 | 2 | 0 | 1 | 1 | 0 | 0 | 0 | 0 | 1 | 1 | 1 | 1 | 1 | **11** |
| Taunton et al. [62] | 2003 | 1 | 1 | 2 | 0 | 1 | 1 | 1 | 1 | 0 | 0 | 1 | 2 | 1 | 1 | 1 | **14** |
| Dane et al. [37] | 2004 | 1 | 1 | 2 | 0 | 0 | 0 | 0 | 0 | 0 | 0 | 1 | 3 | 1 | 1 | 0 | **10** |
| Rauh et al. [57] | 2006 | 1 | 1 | 2 | 1 | 1 | 3 | 1 | 2 | 1 | 2 | 1 | 1 | 1 | 1 | 1 | **20** |
| Plisky et al. [56] | 2007 | 1 | 1 | 2 | 1 | 1 | 3 | 1 | 1 | 1 | 2 | 1 | 3 | 1 | 1 | 1 | **21** |
| Alonso et al. [32] | 2009 | 1 | 1 | 1 | 1 | 1 | 3 | 1 | 3 | 1 | 1 | 1 | 3 | 1 | 2 | 1 | **22** |
| Alonso et al. [33] | 2010 | 1 | 1 | 1 | 1 | 1 | 3 | 1 | 3 | 1 | 1 | 1 | 3 | 1 | 2 | 1 | **22** |
| Buist et al. [7] | 2010 | 1 | 1 | 2 | 1 | 1 | 2 | 1 | 1 | 1 | 2 | 1 | 1 | 1 | 1 | 1 | **18** |
| Alonso et al. [31] | 2012 | 1 | 1 | 1 | 1 | 1 | 3 | 1 | 3 | 1 | 1 | 1 | 3 | 1 | 2 | 1 | **22** |
| Jacobsson et al. [48] | 2013 | 1 | 1 | 2 | 1 | 1 | 2 | 1 | 1 | 1 | 2 | 1 | 1 | 1 | 2 | 1 | **19** |
| Edouard et al. [40] | 2013 | 1 | 0 | 1 | 1 | 1 | 3 | 1 | 3 | 1 | 1 | 1 | 3 | 1 | 2 | 1 | **21** |
| Nielsen et al. [55] | 2014 | 1 | 1 | 2 | 1 | 1 | 3 | 1 | 3 | 1 | 2 | 1 | 3 | 1 | 1 | 1 | **23** |
| Edouard et al. [39] | 2014 | 1 | 0 | 1 | 1 | 1 | 3 | 1 | 3 | 1 | 1 | 1 | 3 | 1 | 2 | 1 | **21** |
| Changstrom et al. [24] | 2015 | 1 | 1 | 2 | 1 | 1 | 2 | 1 | 3 | 1 | 2 | 1 | 1 | 1 | 1 | 0 | **19** |
| Edouard et al. [19] | 2015 | 1 | 0 | 1 | 1 | 1 | 3 | 1 | 3 | 1 | 1 | 1 | 3 | 1 | 2 | 1 | **21** |
| Kluitenberg et al. [50] | 2015 | 1 | 1 | 2 | 0 | 1 | 2 | 1 | 1 | 1 | 2 | 1 | 1 | 1 | 1 | 1 | **17** |
| Hespanhol Junior et al. [45] | 2016 | 1 | 1 | 2 | 1 | 1 | 2 | 1 | 1 | 1 | 2 | 1 | 1 | 1 | 1 | 1 | **18** |
| Hespanhol Junior et al. [44] | 2017 | 1 | 1 | 2 | 0 | 1 | 2 | 1 | 1 | 1 | 2 | 1 | 1 | 1 | 1 | 1 | **17** |
| Rizzone et al. [59] | 2017 | 1 | 0 | 2 | 1 | 1 | 2 | 1 | 3 | 1 | 2 | 1 | 1 | 1 | 1 | 0 | **18** |
| Messier et al. [53] | 2018 | 0 | 1 | 2 | 0 | 1 | 2 | 1 | 1 | 0 | 0 | 1 | 3 | 1 | 1 | 1 | **15** |
| Winter et al. [64] | 2019 | 0 | 1 | 2 | 1 | 1 | 2 | 1 | 2 | 1 | 2 | 1 | 3 | 1 | 1 | 1 | **20** |
| Fokkema et al. [42] | 2019 | 1 | 1 | 2 | 1 | 1 | 2 | 1 | 1 | 0 | 0 | 1 | 1 | 1 | 1 | 1 | **15** |
| Hayes et al. [43] | 2019 | 1 | 1 | 2 | 1 | 1 | 1 | 0 | 0 | 0 | 0 | 1 | 1 | 1 | 1 | 1 | **12** |
| Lagas et al. [51] | 2019 | 1 | 1 | 2 | 0 | 1 | 2 | 1 | 1 | 0 | 0 | 1 | 1 | 0 | 0 | 1 | **12** |
| Ruffe et al. [60] | 2019 | 1 | 1 | 2 | 1 | 1 | 3 | 1 | 2 | 0 | 0 | 1 | 1 | 1 | 1 | 0 | **16** |
| Edouard et al. [41] | 2020 | 1 | 1 | 1 | 0 | 1 | 3 | 1 | 3 | 0 | 0 | 1 | 3 | 1 | 2 | 1 | **19** |
| Winter et al. [65] | 2020 | 1 | 1 | 2 | 1 | 1 | 2 | 1 | 2 | 1 | 2 | 1 | 3 | 1 | 1 | 1 | **21** |
| Hofstede et al. [46] | 2020 | 1 | 1 | 2 | 0 | 1 | 2 | 1 | 1 | 0 | 0 | 1 | 1 | 1 | 1 | 1 | **14** |

**Declarations**

**Funding**

The research fellowship of Karsten Hollander is funded by the German Research Foundation (grant number HO 6214/2-1). No sources of funding were used to assist in the preparation of this article.

**Conflict of interest**

Karsten Hollander, Anna Lina Rahlf, Jan Wilke, Christopher Edler, Simon Steib, Astrid Junge and Astrid Zech declare that they have no conflicts of interest relevant to the content of this review.

**Authorship contributions**

KH: conceptualization, methodology, literature search, risk of bias assessment, formal analysis, writing (original draft preparation)

ALR: conceptualization, methodology, risk of bias assessment, writing (review & editing)

JW: formal analysis, visualization, writing (review & editing)

CE: literature search, writing (review & editing)

SSt: conceptualization, methodology, writing (review & editing)

AJ: conceptualization, methodology, risk of bias assessment, writing (review & editing)

AZ: conceptualization, methodology, literature search, writing (review & editing), supervision

All authors read and approved the final manuscript.
